# High rates of malnutrition and epilepsy: two common comorbidities in children with cerebral palsy

**DOI:** 10.3906/sag-1803-79

**Published:** 2019-02-11

**Authors:** Kürşad AYDIN, Ayşe KARTAL, Esma KELEŞ ALP

**Affiliations:** 1 Department of Pediatric Neurology, Faculty of Medicine, Medipol University, İstanbul Turkey; 2 Department of Pediatric Neurology, Faculty of Medicine, Selçuk University, Konya Turkey; 3 Department of Pediatrics, Dr. Ali Kemal Belviranlı Obstetrics and Children Hospital, Konya Turkey

**Keywords:** Cerebral palsy, malnutrition, epilepsy, children

## Abstract

**Background/aim:**

The aim of this study was to evaluate the nutritional status of children with cerebral palsy and determine the particular characteristics of the disorder.

**Materials and methods:**

The nutritional status of the children was assessed by the Gomez classification using weight-for-age. The Gross Motor Function Classification System was used to determine the gross and fine motor functions.

**Results:**

The study was conducted with 197 children (58.4% males) between the ages of 1 and 18 years old. Asphyxia (44.1%) was the primary etiological factor, and spastic quadriplegia (41.6%) was the most common type of cerebral palsy. Malnutrition was the most frequent comorbidity and the overall malnutrition rate was 76.6%. The most common type of malnutrition was severe malnutrition, which was seen in 70 patients (35.5%). Epilepsy was the second most common comorbidity, seen in 51.7% of the cases.

**Conclusion:**

Our results revealed a high rate of malnutrition and epilepsy in children with cerebral palsy. These two more common significant comorbidities that influence the outcomes of children with cerebral palsy should be carefully evaluated and successfully managed. Families of children with cerebral palsy and their physicians should be educated about the nutritional status in these children.

## 1. Introduction

Cerebral palsy (CP) is a common and chronic neurological disorder occurring in childhood that is caused by permanent and nonprogressive damage of the brain suffered at an early developmental stage (1). The worldwide prevalence of CP is 0.2% of live births, and this rate has been rising in recent years, mainly due to the increase in the survival rate of very-low-birth-weight infants (2–4). Similar to other developing countries, the prevalence of CP is 0.4% in Turkey (5). Cerebral palsy is characterized by abnormal muscle tone, postural control, and motor function. In daily practice, these symptoms are often accompanied by varying degrees of impairments in cognition, perception, behavior, epilepsy, nutritional problems, and secondary musculoskeletal problems (6,7). The presence of these comorbidities depends on the severity of the underlying etiology and the prevention of these problems can have a positive impact on the quality of life in CP patients (8).

Recently, malnutrition has been reported in a considerable number of studies on children with CP. The reasons for malnutrition in children with CP are multifactorial and include both nutritional and other factors. Dietary factors include inadequate nutrient intake as a consequence of gastrointestinal disorders, including oral motor dysfunction, constipation, and gastroesophageal reflux (9). The other factors include the type and severity of the underlying neurological disability, influencing the ambulatory and cognitive status, and antiepileptic drug use (9). The families of children with CP can have insufficient knowledge about proper feeding techniques. Moreover, pediatric neurologists often concentrate on solving the accompanying neurological issues (epilepsy, motor and cognitive difficulties, etc.). Thus, malnutrition in these children is not as frequently recognized and treated as the other associated comorbidities (10). 

Although CP is a common permanent neurological disorder in our country, as in other developed or developing countries, there are not enough studies from Turkey describing the nutritional status of children with CP. Therefore, the current study aimed to examine the nutritional status of a sample of Turkish children with CP. 

## 2. Materials and methods

We retrospectively reviewed the clinical records of 197 patients with CP who were followed at a secondary care pediatric neurological center. These patients were between the ages of 1 and 18 years old. Those with incomplete data during follow-up at the time of evaluation were excluded from the study. Each patient’s chart was reviewed to collect the following clinical information: age, sex, growth parameters (weight, height, and head circumference), maternal age at infant’s birth, gestational age, birth weight, mode of delivery, parental consanguinity, number of newborns, APGAR score or asphyxia history, and neuroimaging findings. The gross motor abilities of the patients with CP were assessed using the Gross Motor Function Classification System (GMFCS). According to this classification, the patients’ motor skills were divided into five levels (mild to severe, respectively), where level I indicates the mildest disabilities and level V the most severe disabilities. 

CP was categorized into four subtypes based on the impairment of gross motor function: spastic (quadriplegia, diplegia, hemiplegia, and monoplegia), dyskinetic, ataxic-hypotonic, and mixed. In the presence of an active history of epilepsy, the age at onset of epilepsy, type and frequency of seizures, number of medications used, nature and localization, and types of electroencephalography (EEG) abnormalities were noted. The seizures, syndrome classifications, and responses to treatment were based on International League Against Epilepsy proposals. Active epilepsy was considered to exist when two or more unprovoked seizures had occurred during the previous year. The patients were classified as having drug-resistant epilepsy when at least two adequate and tolerated antiepileptic drug schedules failed to achieve sustained freedom from seizures.

### 2.1. Anthropometric measurements

The weights of children below 10 kg were measured using a baby scale with 10-g sensitivity (Seca 334, Hamburg, Germany). Children older than 2 years were weighed on an adult scale with 100-g sensitivity (Seca 769, Hamburg, Germany). If a child was unable to stand, the weight was calculated as the difference between the weight of the parent holding the child and the weight of the parent alone. To maintain standard anthropometric measurements, all of the measurements were taken by the same person.

The nutritional status of each child was assessed by the Gomez classification using weight-for-age, which was normal for those between 90% and 110%, mild malnutrition for 75%–89%, moderate malnutrition for 60%–74%, and severe malnutrition for <60%. 

### 2.2. Statistical analysis

All statistical analyses were done using SPSS 17.0 for Windows (SPSS Inc., Chicago, IL, USA). The chi-square test was used to assess the relationships between the categorical variables. Quantitative variables are expressed as mean ± standard deviation, and qualitative variables are given as frequency and percentage. Statistical significance was inferred at P < 0.05. 

## 3. Results

A total of 197 children with a median age of 79.91 ± 49.95 months were enrolled in this study. Of the 197 patients, 82 (41.6%) were girls and 115 (58.4%) were boys. The etiology of CP was hypoxic-ischemic encephalopathy in 87 (44.1%), prematurity in 81 (41.1%), congenital brain malformations in 39 (34%), intrauterine infections in 8 (5%), and kernicterus in 5 (2.5%) patients. The majority of the children had spastic CP. Of these patients, 82 (41.6%) had quadriplegia, 58 (29.4%) had diplegia, 17 (8.6%) had hemiplegia, and 6 (3%) had monoplegia (Table 1). 

**Table 1:** Neurologic findings of 197 children with cerebral palsy.

	Number	%
Sex		
Male	115	58.4
Female	82	41.5
Birth weight		
<2500 g	82	45
2500–3999 g	86	47.8
≥4000 g	13	7.2
Risk factors		
Asphyxia	87	44.1
Prematurity	81	42.1
Cerebral dysgenesis	39	19.7
Intrauterine infections	8	4
Kernicterus	5	2.5
Type of CP		
Spastic	163	82.7
Quadriplegic	82	41.6
Diplegic	58	29.4
Hemiplegic	17	8.6
Monoplegic	6	7.6
Dyskinetic	15	7.6
Hypotonic-ataxic	9	4.6
Mix type	10	5.1
Gross Motor Function Classiﬁcation System level		
Level I	67	36.6
Level II	22	12
Level III	12	6
Level IV	44	24
Level V	38	21.2

Fourteen of the 197 patients were not included in the GMFCS classification because they were under 18 months old. While 67 patients (36.6%) were able to walk unassisted, 38 patients (20.7%) were found to be severely affected. The results were as follows: 21.2% severely impaired (level V), 24% moderately impaired (level IV), 6% mildly impaired (level III), 12% borderline (level II), and 36.6% minimum disability score (level I).

Nutritional status was assessed using the Gomez classification, and the median Gomez score was found to be 75 (range: 29–130). Malnutrition was the most frequent comorbidity and the overall malnutrition rate was 76.6% (151 cases). The most common type of malnutrition was severe malnutrition, which was seen in 70 patients (35.5%); only one in five patients had a normal weight (Table 2). More than half of the patients (58.4%) had difficulties consuming solid food, and they preferred puree (33%) or liquids only (7.1%). The correlation between the type of CP and nutritional status revealed that in the spastic quadriplegic patients the rate of third degree malnutrition (64.3%) was higher than in the other groups, which was statistically significant (P = 0.001, Table 3). On the other hand, no statistical significant was found between birth weight and the severity of malnutrition. However, a relation was found between the level of GMFCS classification and the severity of malnutrition (P = 0.01) which means that while the level of GMFCS classification increased, the severity of malnutrition increased. 

**Table 2:** Malnutrition data of 197 cerebral palsy patients.

	Number	%
3rd degree malnutrition	70	35.5
2nd degree malnutrition	47	23.9
1st degree malnutrition	34	17.3
Normal weight	38	19.3
Overweight	5	2.5
Obese	3	1.5
Total	197	100

**Table 3:** The relationship between clinical subtypes of cerebral palsy and nutritional status. *P = 0.001.

	Number (%)					
Nutritional status	Spasticdiplegia	Spasticquadriplegia	Other spasticand mix types	Hypotonic-ataxictype	Dyskinetictype	Total
3rd degree malnutrition	11 (15.7)	45 (64.3)*	7 (10)	2 (2.9)	5 (7.1)	70 (35.5)
2nd degree malnutrition	15 (31.9)	20 (42.6)	9 (19.1)	1 (2.1)	2 (4.3)	47 (23.9)
1st degree malnutrition	17 (50)	5 (14.7)	6 (17.6)	2 (5.9)	4 (11.8)	34 (17.3)
Normal weight	13 (34.2)	10 (26.3)	7 (18.5)	4 (10.5)	4 (10.5)	38 (19.3)
Overweight	1 (20.0)	1 (20.0)	3 (60.0)	0	0	5 (2.5)
Obese	1 (33.3)	1 (33.3)	1 (33.3)	0	0	3 (1.5)
Total	58	82	33	9	15	197 (100)

### 3.1. Types of seizures and electroencephalographic abnormalities

Of the studied children with CP, 102 (51.7%) had epilepsy, and in the majority of them (63.7%), the seizures had begun before they were 1 year old. More than half of the children with epilepsy (54%) had spastic quadriplegia. The most common seizures were generalized seizures, which were seen in 51 patients (49.8%), with partial seizures in 48 (47.4%) and infantile spasms in three (2.8%) patients. The frequency of EEG abnormalities was 89.2%. Generalized and focal epileptiform discharges were found to be equal in the patients in our study, and both were observed in 39 (38%) patients. In addition, a bioelectric status was found in 6 patients, with hypsarrhythmia in 3 patients. The complete control of seizures was achieved in 74 patients (72.6%), with partial control in 14 patients (13.7%), while 14 patients (13.7%) exhibited poor or no response to either older or newer generations of antiepileptic drugs. Forty-seven (49%) patients were using only one antiepileptic drug (AED), 33.7% used two AEDs, and 17.3% used three or more AEDs. Only five patients did not use any AEDs. The relationship among the subtype of CP, the accompanying epilepsy, and seizure control was not statistically significant.

## 4. Discussion

According to the Surveillance of Cerebral Palsy in Europe, speech and language impairments, severe intellectual disability, epilepsy, and visual impairment are the most common comorbidities, respectively (1). We found a higher prevalence of speech and language impairments and malnutrition than indicated in earlier reports from both high-income and other low-income countries (11–13). However, this study showed us some new and important findings in children with CP in Turkey. Malnutrition was the most common and most important comorbidity in these children, and the prevalence of malnutrition was found to be 76.7% overall. Furthermore, among the malnourished patients, third degree malnutrition was the most commonly observed type of nutrition. As expected, many of these patients had quadriplegia. One study from Turkey reported that the rate of malnutrition in severely disabled patients followed by two different pediatric neurology departments were 72% and 64%, respectively (11). A strong association between malnutrition and the subtype of CP has been reported (11). Additionally, malnutrition has a multifactorial etiology in children with CP. As expected, a lower level of motor ability was associated with an increased risk of feeding and swallowing problems (9,10). 

Numerous factors are known to influence nutrition in children with CP and can be explained as follows. First, because these patients have poor motor control and increased muscle tone, sucking and swallowing difficulties, drooling, food refusal, and difficulty consuming solid food are common (1–3). Second, prolonged meal times are stressful for the families of children with CP, which increases the time needed for meals and decreases the effectiveness of unassisted eating (11). Therefore, most families have been restricting the diets, especially solid and grained food, in children with CP. Furthermore, previous studies revealed that malnutrition during childhood was related to delayed mental and psychomotor development, in addition to behavior problems, such as attention deficit disorders and aggressive behavior (14). This situation causes a further increase in the nutritional problems in children with existing mental and behavioral problems. Overall, the presence of malnutrition in children with CP causes adverse effects on the quality of life in these children.

There are some alternative ways to improve nutrition in children with CP, such as gastrostomy tube feeding. Some studies have revealed that gastrostomy tube feedings are safe and efficient for nutritional support (15). However, in our study, we found that only one child was fed by a gastrostomy tube. Gastrostomy tube feeding was recommended for those patients with severe dysphagia and malnutrition, but the parents did not accept this procedure. It is very important to discuss the decision to perform a gastrostomy with the family, since it will individualize the positive and adverse effects.

Whatever the reason for malnutrition, it develops over a long period of time. These children with CP were repeatedly seen by pediatricians or pediatric neurologists during the follow-up time. Unfortunately, the physicians often concentrated on solving the neurological problems, and malnutrition was not as frequently recognized and treated as the other associated comorbidities (10). Therefore, the awareness of physicians about malnutrition in children with CP should be increased.

The reported prevalence of epilepsy in children with CP is variable, ranging from 33% to 62% (16,17). We found this prevalence as 51.7% of the children with CP in our study. In most of them (63.7%), the seizures began before they were 1 year old, unlike the known reports. The frequency of epilepsy varies according to the subtype of CP, and it has been reported to occur most commonly in the quadriplegic type (18,19). Consistent with these studies, we found a high incidence of epilepsy in our quadriplegic patients (59.4%). Polytherapy was required in half of the patients, and 13.7% of these patients had refractory epilepsy. The presence of epilepsy and seizure control is also related to the type of CP. However, we did not find any relationship between the subtypes of CP and these parameters. The frequency of epilepsy in children with CP was high and the majority of the patients achieved complete seizure control with monotherapy.

In conclusion, our study reported a higher rate of malnutrition and epilepsy in children with CP. These most common comorbidities were high in those children with quadriplegic CP. Certainly, many factors can cause malnutrition, such as inadequate food intake and neurological and gastrointestinal problems, and these must be eliminated in these children. However, the awareness of families and physicians about the nutritional status of children with CP should be increased.
